# Mutagenesis on a complex mouse genetic background by site-specific nucleases

**DOI:** 10.1007/s11248-024-00399-5

**Published:** 2024-08-01

**Authors:** Benjamin Davies, Lucy Trelfa, Victoria S. Rashbrook, Edward Drydale, Rachel Martin, Boyan Bai, Jedrzej Golebka, Daniel Stephen Biggs, Keith M. Channon, Shoumo Bhattacharya, Gillian Douglas

**Affiliations:** 1 Division of Cardiovascular Medicine, Radcliffe Department of Medicine, BHF Centre of Research Excellence, John Radcliffe Hospital, University of Oxford, Oxford, UK; 2 Wellcome Centre for Human Genetics, University of Oxford, Roosevelt Drive, Oxford, UK; 3 Francis Crick Institute, 1 Midland Road, London, UK

**Keywords:** 3Rs, Atherosclerosis, Regression, Site directed mutagenesis, *Abcg1*

## Abstract

Mouse models with complex genetic backgrounds are increasingly used in preclinical research to accurately model human disease and to enable temporal and cell-specific evaluation of genetic manipulations. Backcrossing mice onto these complex genetic backgrounds takes time and leads to significant wastage of animals. In this study, we aimed to evaluate whether site-specific nucleases could be used to generate additional genetic mutations in a complex genetic background, using the REVERSA mouse model of atherosclerosis, a model harbouring four genetically altered alleles. The model is comprised of a functional null mutation in the *Ldlr* gene in combination with a *ApoB100* allele, which, after high-fat diet, leads to the rapid development of atherosclerosis. The regression of the pathology is achieved by inducible knock-out of the *Mttp* gene. Here we report an investigation to establish if microinjection of site-specific nucleases directly into zygotes prepared from the REVERSA could be used to investigate the role of the ATP binding cassette transporter G1 (ABCG1) in atherosclerosis regression. We show that using this approach we could successfully generate two independent knockout lines on the REVERSA background, both of which exhibited the expected phenotype of a significant reduction in cholesterol efflux to HDL in bone marrow-derived macrophages. However, loss of *Abcg1* did not impact atherosclerosis regression in either the aortic root or in aortic arch, demonstrating no important role for this transporter subtype. We have demonstrated that site-specific nucleases can be used to create genetic modifications directly onto complex disease backgrounds and can be used to explore gene function without the need for laborious backcrossing of independent strains, conveying a significant 3Rs advantage.

**Supplementary Information:**

The online version contains supplementary material available at 10.1007/s11248-024-00399-5.

## Introduction

Genetic mouse models are essential tools for understanding gene function of complex diseases. Accurately modelling human genetic disease in the mouse can necessitate multiple genetic perturbations or combinations of alleles to increase the translational relevance of the animal model. Genetically altered alleles are traditionally combined by simple interbreeding of parental mouse strains. However, due to the Mendelian segregation of alleles, this becomes a very time consuming and inefficient process as the number of alleles increases. Most importantly, a large number of surplus animals with non-useful combinations of alleles are generated. Indeed, in recent years, the breeding of genetically altered animal models constitutes a large proportion (45%) of the total number of regulated animal procedures performed in biomedical research (Home Office 2022). As technology progresses, new tools have become available to achieve both temporal and cell specific manipulations of genes. Invariably, these techniques require additional genetic alleles to achieve this level of control, adding to the breeding complexity issues associated with multiple genetic alleles.

The advent of site-specific nucleases has allowed the direct manipulation of genetic alleles within the zygote, through the introduction of these gene editing tools by microinjection or electroporation into the preimplantation embryo. Such techniques have the potential to allow the genetic manipulation of a pre-existing complex genetic background, thus circumventing the requirement for complex and lengthy interbreeding. This approach has considerable 3Rs potential, as it could avoid the generation of surplus animals with undesirable genotypes that result from traditional interbreeding approaches. In addition, it could also offer a significant decrease in the time taken to generate additional genetic manipulations on complex backgrounds.

Atherosclerosis is a chronic inflammatory disease of the vessel wall driven by high circulating low density lipoprotein (LDL) levels, endothelial cell activation and inflammatory cell recruitment. Although historically atherosclerosis was considered an irreversible condition, advancement in pre-clinical atherosclerosis regression models and imaging technologies in clinical trials have revealed that atherosclerosis is a dynamic disease state that can regress, progress or stabilise (Underhill et al. 2008). However, one of the major limitations to understanding the cellular mechanisms of atherosclerosis regression has been the availability of suitable animal models.

One such mouse model is the REVERSA mouse (*Ldlr*^*−/−*^; *Apob*^*100/100*^; *Mttp*^*fl/fl*^; *Mx1Cre*^+*/*+^) which is a model of human familial hypercholesterolemia driven by loss of the LDL receptor and expression of *Apob100* (Lieu et al. 2003). Additionally, in this mouse model, cholesterol levels can be normalized by the induction of the Mx1-cre transgene (by polyI-polyC (pI:pC) injection) resulting in excision of *Mttp* (required for ApoB secretion) leading to a fall in plasma LDL and VLDL levels and atherosclerosis regression (Lieu et al. 2003), with loss of monocyte-derived cells from the plaque (Feig et al. 2011a; Parathath et al. 2011). This model thus allows an exploration of the mechanisms of atherosclerosis progression and regression.

The complex genetic make-up of this model, however, does not lend itself to further genetic perturbation. Crossing this mouse model to a conventional knock-out, generated on a standard wild-type inbred strain background, would necessitate at least 5 generations of breeding to re-establish the genetic characteristics of the model background, resulting in an unacceptably large animal usage and several years of breeding.

As an alternative, site-specific nucleases, such as CRISPR/Cas9 or Transcription activator-like effector nucleases (TALENs), can be used to ablate gene function directly on zygotes prepared from such a complex genetic model. Previously, double knock-out models and pre-existing disease backgrounds have been used successfully for further genetic manipulation (Davison et al. 2018; Du et al. 2019). Here we sought to explore an extreme case of whether a 5th gene could be manipulated directly in zygotes prepared form the complex REVERSA genetic background harbouring 4 pre-existing homozygous alleles by TALEN microinjection. We have explored the role of a candidate gene involved in reverse cholesterol transport, *Abcg1*.

ATP binding cassette transporter A1 (ABCA1) and G1 (ABCG1) are transmembrane transporters that export cellular lipids to plasma acceptors such as HDL and ApoA-I. Together, they constitute a key component of reverse cholesterol transport (RCT) pathway and a major mechanism for the regulation of cellular cholesterol in non-hepatic tissues. Clinically, HDL-mediated cholesterol efflux from macrophages has been shown to be inversely associated with carotid intima-media thickness, independently of serum HDL levels (Khera et al. 2011), implicating RCT as a negative regulator of plaque size. In addition, murine surgical regression models have supported a role of HDL, with HDL shown to promote CD68 positive cell cholesterol efflux from atherosclerotic plaques (Feig et al. 2011b).

Clinical and pre-clinical evidence suggest that ABCG1 has a role potential role in atherosclerosis. Patients with atherosclerosis have been shown to have significantly reduced ABCG1 levels in circulating peripheral blood mononuclear cells (Rafiei et al. 2021). Genetic ablation of *Abcg1* results in a shift in macrophage phenotype from a M2 (pro-resolution) to a M1 (pro-inflammatory) phenotype (Sag et al. 2015) with increased pro-inflammatory signalling via toll like receptors (TLR), in particular TLR4 (Yvan-Charvet et al. 2008) which would promote atherosclerosis progression and inhibit atherosclerosis regression which is promoted by pro-resolution M2 like macrophages. Genetically the association of *ABCG1* with coronary artery disease have been mixed. Two SNPs, found in the regulator region of *ABCG1* (rs1893590 and rs1378577), were shown to have no impact on angiographic outcomes, however, both SNPs were associated with changes in LPL activity (Olivier et al. 2012). In addition, the rs57137919 SNP, which is associated with increased apoptosis in cholesterol loaded macrophage, is associated with a reduction in coronary artery disease (Xu et al. 2011). A protective role for *ABCG1* in coronary artery disease has been observed in the Copenhagen City Heart Study with three variants found to be associated with myocardial infarction (Schou et al. 2012). However, no study to date has specifically investigated the role of ABCG1 in atherosclerosis regression.

Here we use TALEN mutagenesis to successfully engineer loss-of-function alleles directly on the REVERSA background. In contrast to the previous studies using the classical *Abcg1* knock out model, this complex genetic model allows an exploration of how a loss of *Abcg1* and resulting changes in RCT affect plaque regression and showcases the use of genomic editing on complex genetic backgrounds.

## Results

### Generation of *Abcg1* knock out mice on the REVERSA background

**Fig. 1:**
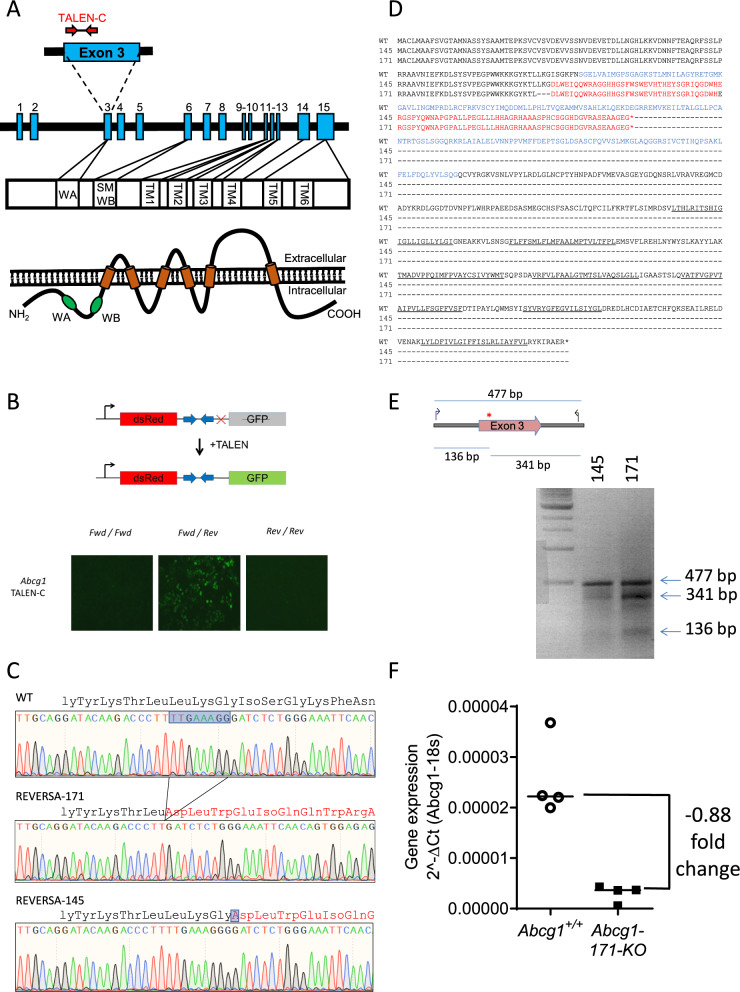
Generation of *Abcg1* knock out mice on the REVERSA background. **a** Structure of the *Abcg1* gene and the domain structure of the ABCG1 protein, showing Walker A (WA), Walker B (WB), signature motif (SM) and transmembrane domains (TM). The target binding sites of the two TALEN monomers within exon 3 are shown. **b** Surrogate reporter assay with the target site for the TALEN cloned between a dsRed expression cassette and an out-of-frame GFP cassette. Cleavage of the target site by the functional TALEN pair (Fwd/Rev) results in indel mutations which restores the reading frame, allowing GFP expression. Introduction of the two monomers (Fwd/Fwd and Rev/Rev) serve as a control. **c** Chromatographs of the wild-type and mutant lines under analysis, showing the 1 bp insertion (line 145) and 8 bp deletion (line 171). The impact on the open reading frame is shown above the sequence detail and amino-acid residues differing from the wild-type ABCG1 protein are shown in red. **d** Predicted protein sequences of the mutant alleles, aligned against the wild-type ABCG1 protein. Both mutant alleles are missense mutations (differences shown in red) which result in significantly truncated protein (devoid of ATPase domain (blue) and the 7 transmembrane domains of the transporter (underlined). **e** Genotyping of the mutant alleles, showing the resulting of a Cel1 nuclease assay which cleaves the *Abcg1* amplicon at the position of the introduced mutation. **f** Real time qRT-PCR showing a significant reduction (*P* < 0.05, *T* Test, n = 4 per group) in the expression of *Abcg1* in the liver of *Abcg1*^*−/−*^ mice compared with *Abcg1*^+*/*+^ mice

Murine *Abcg1* comprises 15 exons that gives rise to a protein containing 6 transmembrane domains and an ATP binding cassette, comprising the Walker A, Walker B and signature motif (Fig. 1a). We used TALENs to generate an *Abcg1*-Knock-out REVERSA mouse, using techniques that we have previously established for high-efficiency TALENs-mediated genome editing in the mouse (Davies et al. 2013). TALEN enzymes were designed against *Abcg1* exon 3 (Fig. 1a), which is common to all transcripts and contains the phosphate binding Walker A domain necessary for ATPase activity (Vaughan and Oram 2005), and were validated for activity in a surrogate reporter assay (Davies et al. 2013) (Fig. 1b). *Abcg1* TALEN mRNA was injected in the cytoplasm of REVERSA oocytes and the resulting embryos were transferred to foster mice and allowed to develop to term. Three founder lines with mutations were generated (*Abcg1*-145, harbouring a single nucleotide insertion, *Abcg1*-752, harbouring a 9 nucleotide deletion and *Abcg1*-171, harbouring a 8 nucleotide deletion). The mutant alleles present in *Abcg1*-145 and -171 contain frame-shift mutations within the phosphate binding Walker A domain, resulting in the insertion of a premature stop codon. The resulting missense peptide sequences in both lines lead to a loss of both the ATPase domain and transmembrane domains (Fig. 1c, d). The Walker A domain is essential for activity of ABCG1 with mutation of only a single glycine residue with the Walker A motif sufficient to abolish the ability of ABCG1 to redistribute cholesterol (Vaughan and Oram 2005). In addition, no splice variants which exclude the mutated exon are known to exist and any aberrant splicing around the mutation would result in a non-functioning protein due to the loss of the essential catalytic domain. Given this evidence of both of these lines were considered likely to harbour null alleles and were taken forward for further analysis. Experiments to confirm the knock-out at the protein level by both Western blot analysis and immunohistochemistry of liver samples using ABCG1-specific antibodies failed to yield conclusive results as the specificity of the antibodies could not be confirmed in wild type tissue. Both mutations were nonetheless considered to be functional null alleles because the predicted missense peptide sequences would be extremely truncated (Fig. 1d) and would fail to embed into the membrane and would lack the key ATPase domain necessary for activity. Cel1 nuclease assay which cleaves the *Abcg1* amplicon at the position of the introduced mutations was used to confirm the genotypes of the founders (Fig. 1e). These founders were bred with REVERSA mice to generate mice heterozygous for the *Abcg1* knock-out mutations, but with the REVERSA background alleles preserved homozygously. These F1 *Abcg1* KO heterozygous mice were then bred together to generate homozygotes (hereafter referred to as *Abcg1*-171-KO and *Abcg1*-145-KO).

RNA extracted from primary macrophages from wild-type REVERSA (hereafter referred to as *Abcg1*^+/+^) mice and mice homozygous for the *Abcg1*-145 and -171 mutations was used to confirm that the mutations were transcribed to mRNA. Intron-spanning primers were designed, a product of the expect size was obtained and sequence analysis confirmed the insertion (*Abcg1*-145) and deletion (*Abcg1*-171) within the Walker A domain of *Abcg1*. The expression level of *Abcg1* were also assessed by quantitative RT-PCR and this revealed a significant reduction in mRNA expression of *Abcg1* in the livers of *Abcg1*-171-KO mice compared with their *Abcg1*^+/+^ littermates (Fig. 1f), suggestive of nonsense mediated decay mechanisms reducing the levels of mutant transcript.

### Validation of *Abcg1* knock out mice on the REVERSA background

**Fig. 2:**
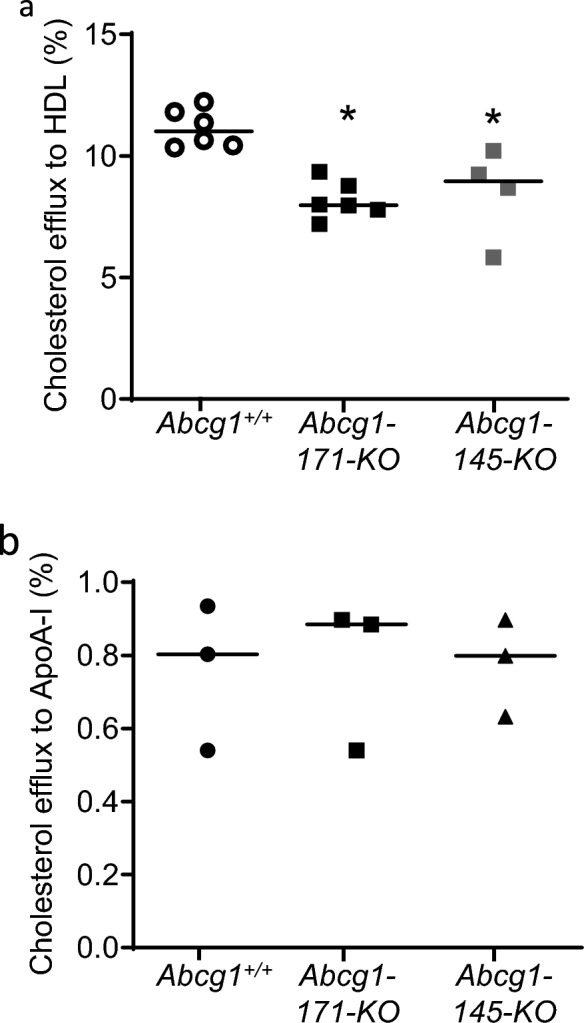
Loss of *Abcg1* resulted in a significant reduction in cholesterol efflux to HDL. A significant reduction in cholesterol efflux to HDL (50 µg/ml; derived from plasma) was observed in bone marrow derived macrophages from both *Abcg1*^−/−^ lines (* *P* < 0.05, one way ANOVA *Abcg1*^−/−^ to *Abcg1*^+/+^). Data are expressed as the mean ± SEM, each data point represents an individual animal

As discussed above, despite the commercial availability of ABCG1 antibodies, we were unable to obtain a definitive result in wild-type tissue, despite testing numerous conditions, making it impossible to validate our putative knock-out alleles using standard Western blotting approaches. Instead, we adopted a functional test to demonstrate that the targeted *Abcg1* alleles resulted in the expected loss of ABCG1 function. We performed a cholesterol efflux assay in bone marrow-derived macrophages from REVERSA *Abcg1*^+/+^ mice and both the *Abcg1*-145-KO and the -171-KO models. As demonstrated in previous studies in *Abcg1*-KO mice (Wang et al. 2007) and in cell lines with siRNA knock down of *Abcg1* (Wang et al. 2004), both putative *Abcg1* knock-out lines exhibited a significant reduction in cholesterol efflux to HDL (purified from plasma), with no difference observed between the two independent lines (Fig. 2). Critically, the magnitude of the reduction was almost identical to published values for the *Abcg1*^−/−^ *ApoE*^−/−^ mice (Wang et al. 2007), confirming the validity of the TALEN-induced gene knock-out. As both lines showed a similar degree of inhibition of *Abcg1* function we decided to take forward the *Abcg1*-171-KO line, hereafter referred to as *Abcg1*^−/−^ for further analysis.

### Loss of *Abcg1* did not impact on atherosclerosis progression or regression

**Fig. 3:**
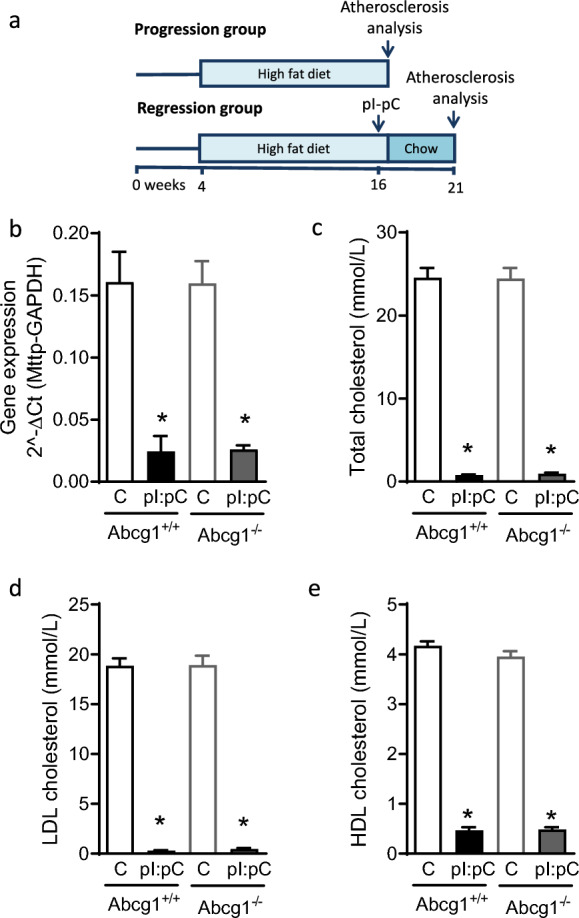
Loss of *Abcg1* did not alter the circulating lipid profile either before or after pI:pC injection to induce normalization of lipid levels. **a** Schematic diagram of the study time line. **b** pI:pC injection lead to a significant reduction in *Mttp* expression in the liver (*P ≤ 0.05, two way ANOVA comparing pI:pC treatment with respective controls) with no difference observed between genotypes either before or after pI:pC injection (*P* > 0.05, one way ANOVA). pI:pC injection lead to a significant reduction in plasma levels of total cholesterol (**c**), LDL cholesterol (**d**) and HDL cholesterol (**e**); *P ≤ 0.05, two way ANOVA comparing pI:pC treatment with respective controls). No difference was observed between genotypes either before or after pI:pC injection (*P* > 0.05, one way ANOVA). Data are expressed as the mean ± SEM

In order to investigate the role of *Abcg1* in atherosclerosis progression and regression, *Abcg1*^+/+^ and *Abcg1*^−/−^ mice on the REVERSA background were fed a high fat diet for three months from 4 to 16 weeks of age to induce atheroma. In order to compare atherosclerosis progression between *Abcg1*^+/+^ and *Abcg1*^−/−^ mice, a cohort of mice were harvested at 16 weeks prior to regression. The remaining mice were injected with pI:pC (Ip 15 mg/kg 4 × every other day) to induce *Mttp* deletion, normalization of lipids levels and atherosclerosis regression and maintained for a further 4 weeks (Fig. 3a).

As expected pI:pC injection resulted in a significant reduction in *Mttp* expression in the liver of both *Abcg1*^+/+^ and *Abcg1*^−/−^ mice, with no difference in *Mttp* expression levels observed between the two genotypes either before or after pI:pC administration (Fig. 3b). Loss of *Abcg1* did not result in a significant difference in either total, LDL or HDL cholesterol. After administration of pI:pC a significant reduction in plasma lipid levels was observed in both groups with no difference observed in either total, LDL or HDL cholesterol between the two genotypes (Fig. 3c–e).

**Fig. 4:**
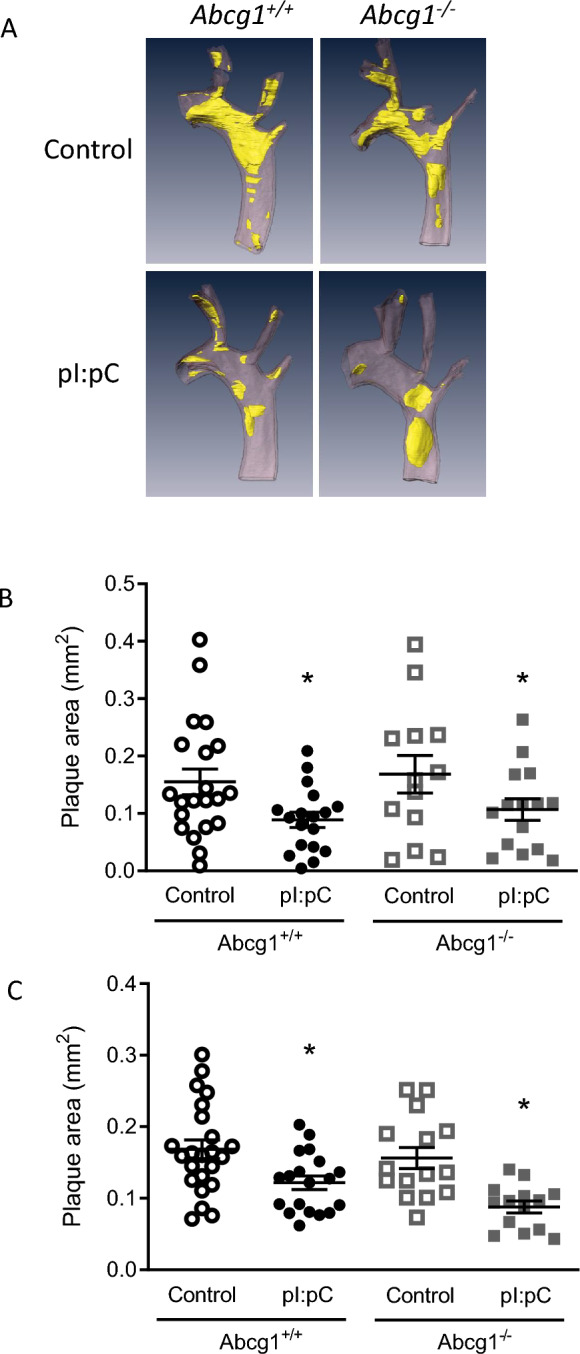
Loss of *Abcg1* does not impact atherosclerosis regression or progression. **a** Representative three-dimensional renders generated from microCT images of atherosclerosis plaques. pI:pC injection and the resultant normalization of lipid levels resulted in a significant reduction in plaque area in both *Abcg1*^+/+^ and *Abcg1*^−/−^ mice. However, no effect of *Abcg1* genotype was observed either before or after pI:pC injection (*P > 0.05, two way ANOVA comparing pI:pC treatment with respective controls) in either the aortic arch (**b**) or the aortic root (**c**). Data are expressed as the mean ± SEM, with each data point representing an individual mouse. Black symbols = *Abcg1*^+/+^, grey symbols = *Abcg1*^−/−^. Open symbols atherosclerosis progression study harvested at 16 weeks of age. Closed symbol regression study harvested at 20 weeks of age 4 weeks after pI:pC injection

To assess the impact of loss of *Abcg1* on plaque progression we harvested a cohort of mice after 16 weeks of high fat diet. At this time point loss of *Abcg1* did not impact plaque progression with no difference in plaque area observed between the two genotypes in either the aortic arch (Fig. 4a, b) or the aortic root (Fig. 4c). Normalization of lipid levels by pI:pC injection resulted in a significant reduction in atherosclerosis plaque area in both the aortic arch and the aortic root (*P* < 0.05, Two way ANOVA). However, loss of *Abcg1* did not appear to impact atherosclerosis regression with no significant difference in plaque area observed between the two genotypes in the aortic arch or root (Fig. 4b, c). As expected, normalization of lipid levels resulted in a significant reduction in plaque macrophage content as assessed by Galectin-3 (MAC-2) positive staining and a significant increase in plaque collagen content (Sirius red staining) in both genotypes (*P* < 0.05, Two-way ANOVA, Supplementary Fig. S1) but there was no difference between genotypes in either the progressive or regressive environment.

## Discussion

In this study we successfully used TALEN site-specific nucleases to knock out *Abcg1* directly on the complex genetic background of the REVERSA mouse—a model comprised of four individual homozygous alleles. Using this complex knock-out mouse we show that loss of *Abcg1* leads to the expected reduction in cholesterol efflux to HDL but that this did not impact circulating cholesterol levels or plaque area in either a progressive or regressive atherosclerosis environment.

The study showcases one of the key advantages of site-specific nuclease technology for the production of mutant alleles—the ability to modify the genome directly in the zygote. Since zygotes can be prepared from any mouse strain, including models with unique combinations of alleles modelling a specific disease and, in general, mice with any pre-existing genetically altered alleles. Previous work has shown the use of CRISPR/Cas9 nucleases to introduce a 3rd mutation on a complex immunodeficient NSG model harbouring 2 pre-existing genetic mutations in the *Prdkc* and *Il2rg* genes (Du et al. 2019). Our study extends these ideas to achieve site-specific mutation of a 5th genetic allele (*Abcg1*) on a model, already harbouring 4 genetically modified loci (*Ldlr*^*−/−*^; *Apob*^*100/100*^; *Mttp*^*fl/fl*^; *Mx1Cre*^+*/*+^). The work thus presents a significant 3Rs advance, as with 4 homozygous genetic alleles, introducing a mutation of a 5th gene by conventional breeding would necessitate lengthy and complex backcrossing and the generation of a large surplus of mice with undesirable genotypes.

One potential weakness of our study is the lack of evidence at the protein level concerning the loss-of-function induced by the frameshift mutations. Unfortunately, the available antibodies tested yielded only non-specific signals, and, importantly, failed to unambiguously detect ABCG1 protein in wild-type mice, making it impossible to conclude. We are, however, confident that our mutations induce functional null alleles based on three lines of evidence. Firstly, at the genomic level, the frameshift mutations are present within the phosphate binding Walker A domain necessary for ATPase activity, with mutation of only a single glycine residue with the Walker A motif sufficient to abolish the ability of ABCG1 to redistribute cholesterol (Vaughan and Oram 2005). There are no known splice variants which exclude the mutated exon, and any aberrant splicing around the mutation that might be conceivably occurring would still be expected to produce a functionally null protein, as the essential catalytic domain would be excluded. Secondly, at the transcript level, we observe a significant reduction in transcript levels in primary macrophages, suggesting nonsense mediated decay as a result of the induced premature stop codons is occurring. Thirdly, at a functional level, we confirm that bone marrow-derived macrophages from mutant mice show a significant reduction in cholesterol efflux to HDL, at a level entirely consistent with the previously published *Abcg1* knockout models (Wang et al. 2007) and with data from *Abcg1* knock-down cell lines (Wang et al. 2004).

In our study we show no impact of loss of *Abcg1* on atherosclerosis regression. Macrophages are a hallmark of atherosclerosis contributing to foam cell formation and local inflammation, thus it is unsurprising that increased macrophage egress and phenotype switching to a pro-resolution M2-like phenotype is associated with increased regression (Feig et al. 2011c; Cardilo-Reis et al. 2012; Ramsey et al. 2014). Loss of *Abcg1* in macrophages leads to an increase in pro-inflammatory cytokines such as IL-6 and IL-1*β* and a decrease in anti-inflammatory cytokines such as IL-10 (Wojcik et al. 2008), promoting an pro-inflmamatory M1 polarization (Sag et al. 2019), as well as increased macrophage apoptosis (Wojcik et al. 2008; Tarling et al. 2010). In addition, deficiency in *Abcg1* in endothelial cell leads to a pro-atherogenic phenotype with reduced NO production (Terasaka et al. 2010). Taken together this would suggest that loss of *Abcg1* in our current study would be expected to result in a pro-inflammatory macrophage phenotype and decreased atherosclerosis regression. However, in our current study we observed no difference in atherosclerosis progression or regression in either plaque area or composition. It is possible that in our study, the ubiquitous loss of *Abcg1* lead to a pro-atherogenic effect in macrophages that was counterbalanced by an enhanced Treg mediated atherosclerosis regression, resulting in no overall change in plaque area. In contrast to *Abcg1* deficiency in macrophages, loss *Abcg1* in T cells and in particular Tregs has been found to be protective against atherosclerosis progression (Cheng et al. 2016). Tregs have been shown to have a key role in atherosclerosis regression by altering the balance of effector T cells and the M1:M2 ratio in favour of a pro-resolution M2 phenyotype (Sharma et al. 2020).

Atherosclerosis progression studies in *Abcg1*^*−/−*^ mice have been mixed, a comprehensive review of atherosclerosis studies in *Abcg1*^*−/−*^ mice on an LDL^−/−^ background shows that in mice loss of *Abcg1* in early lesions leads to enhanced progression. However, in late disease a decreased expression of *Abcg1* leads to reduced atherosclerosis due to increase macrophage apoptosis (Meurs et al. 2012). Clinically this is mirrored by the rs57137919 SNP which is associated with reduced coronary artery disease (Xu et al. 2011) and increase apoptosis in cholesterol loaded macrophages. From this data it is clear to see that *Abcg1* has multiple contrasting roles in atherosclerosis which taken together along with our findings indicate that *Abcg1* is not a rational target in atherosclerosis progression or regression.

In conclusion, we show that TALEN site-specific nucleases can be used to create genetic modifications directly onto complex genetic backgrounds in a single generation, avoiding complex interbreeding. Using this technology, we show that global loss of *Abcg1* does not alter atherosclerosis regression.

## Materials and methods

### Generation of *Abcg1* knock out mice

Plasmids encoding two TALEN enzyme monomers were constructed by Golden Gate assembly of repeat-variable di-residue domain into pTAL3 using the Golden Gate TALEN and TAL Effector kit (Addgene #1,000,000,016). The TALEN was designed against the sequence 5′- tgcaggatacaagaccct-3′ for the sense strand (Fwd) using the RVD array NG-NN-HD-NI-NN-NN-NI-NG-NI-HD-NI-NI-NN-NI-HD-HD-HD-NG and 5′- tctccactgttgaatt-3′ on the antisense strand (Rev) using the RVD array NG-HD-NG-HD-HD-NI-HD-NG-NN-NG-NG-NN-NI-NI-NG-NG. The TALEN arrays were subcloned into pcDNA3 (Life Technologies) and validated for activity in a surrogate reporter assay (Davies et al. 2013). Having confirmed appreciable activity, the pcDNA3-TALEN vectors encoding both the sense and antisense monomer were linearized and used for in vitro transcription from the T7 RNA polymerase promoter using the mMessage mMachine T7 Kit (Life Technologies) according to the manufacturer’s instructions. The resulting mRNAs were purified using the MEGAclear kit (Life Technologies), and eluted in microinjection buffer (1 mM Tris.HCl pH7.5/0.1 mM EDTA).

REVERSA female mice at 21–28 days of age (*Ldlr*^*−/−*^*; Apob*^*100/100*^*; Mttp*^*fl/fl*^*; Mx1Cre*^+*/*+^ (Lieu et al. 2003); Jax stock No: 004192) were superovulated and mated with REVERSA stud males, and fertilized zygotes harvested from plugged females at 0.5 dpc. TALEN mRNAs were diluted to 5 ng/ul in microinjection buffer and microinjected into the cytoplasm. After overnight culture, the resulting two-cell embryos were transferred surgically to pseudopregnant CD1 foster mothers at 0.5 dpc.

Putative founders were identified by amplifying a 409 bp region of the *Abcg1* gene encompassing the TALEN target site, using the primers (Abcg1-F1: 5′-aagcaagatgcatgtgccct-3′; Abcg1-R1: 5′-gcaaaccgttgacaaaatgt-3′) followed by the Cel1 nuclease assay (Surveyor MDK kit, Transgenomic), according to the manufacturer's instructions, and Sanger sequencing. Mutant founder mice were bred with REVERSA mice to maintain the complex genetic background and transmit the *Abcg1* mutations through the germline.

### Atherosclerosis study design

The generation and phenotyping of the knock-out model was carried out in accordance with the Animal [Scientific Procedures] Act 1986, with procedures reviewed by the clinical medicine animal care and ethical review body (AWERB), and conducted under project licenses PPL 30/2562 and 30/3080. All procedures conformed to the Directive 2010/63/EU of the European Parliament. Animals were housed in individually ventilated cages (between 4 and 6 mice per cage of mixed genotypes) in specific pathogen free conditions. All animals were provided with food and water ad-libitum and maintained on a 12 h light: 12 h dark cycle at controlled temperature (20–22 °C) and humidity.

Female *Abcg1*^−/−^ REVERSA (referred to as *Abcg1*^*−/−*^) mice and their *Abcg1*^+/+^ REVERSA littermates (referred to as *Abgc1*^+*/*+^*)* were fed a high fat diet (HFD; SDS 829108 Western RD diet), containing 20% fat, 0.15% cholesterol, for three months from 4 to 16 weeks of age. In order to compare the magnitude of regression observed (percentage regression) in *Abcg1*^−/−^ REVERSA mice to that of their *Abcg1*^+/+^ REVERSA littermates a cohort of mice were harvested at 16 weeks prior to regression. The remaining mice were injected with pI:pC (Ip 15 mg/kg 4 × every other day) to induce atherosclerosis regression by excision of loxP sites flanking *Mttp*. Mice were maintained for a further 4 weeks on a standard chow diet before harvest at 20 weeks of age.

### Tissue collection

All mice were culled by exsanguination under terminal anaesthetic (isoflurane > 4% in 95% O_2_ 5% CO_2_); depth of anaesthesia was monitored by respiration rate and withdrawal reflexes. Biochemical analyses of plasma lipids were performed on heparinised blood plasma using enzymatic assays. Tissue for histological analysis was collected from mice perfused with phosphate buffer saline (PBS) followed by 4% paraformaldehyde, tissue for biochemical analysis was collected from mice perfused with PBS only and was snap frozen in liquid nitrogen and stored at − 80 °C until analysis.

Total RNA was extracted using the Ambion Pure Link kit, RNA was reverse transcribed using the QuantiTech reverse transcription kit (Qiagen), quantitative real-time RT-PCR was performed with an iCycler IQ real-time detection system (BioRad Laboratories). Gene expression data were normalized to an appropriate house keeper using the delta CT method.

### Cholesterol efflux assay

Bone marrow derived macrophages (BMDM) were obtained as follows. Bone marrow was obtained by flushing the femur and tibia of adult mice with PBS. A single cell suspension was prepared by passing the bone marrow through a 70 µm cell strainer. Cells were cultured in 10 cm non tissue culture treated dishes for 7 days in DMEM:F12 (Invitrogen) supplemented with 100 U/ml penicillin and 100 ng/ml streptomycin (Sigma), 10% (v/v) fetal bovine serum (PAA Laboratories), 5 mM L-glutamine (Sigma), and 10–15% (v/v) L929 conditioned medium. Following differentiation, cells were harvested and plated into 6- plates in Optimem with 0.2% fatty acid free serum and labelled with [3H] cholesterol (2 µCi/ml) for 16 h. Cells were washed and left for a further 16 h to equilibrated and cholesterol efflux to HDL cholesterol derived from human plasma (50 µg/ml; Bioquote J64903) assessed after 6 h by scintillation counting of media. Total cellular [H3] cholesterol was determined by extraction of cellular lipids by isopropanol and measurement by scintillation counting. Cholesterol efflux was determined by dividing the radioactive content of the media by the sum of the radioactivity in the cells and the media. The basal efflux to media was subtracted from the values obtained in the presence of HDL.

### Analysis of atherosclerosis

Lesion size was assessed in frozen sections of aortic root stained with Oil Red O (Sigma). Average lesion size was calculated from 6 sections taken at 45 µm intervals starting from the section showing all three aortic valve cusps. Macrophage (Gal-3; R&D systems AF1197, 1/250; detected using the Vector red substrate kit; SK-5100) and collagen (Picro Sirius Red staining) areas were quantified from digitized microscopic images using Image-Pro Plus.

3D atherosclerotic plaques in the aortic arch were visualised using microCT. Arches were dissected free of fat and connective tissue and incubated in 2% phosphotungstic acid in PBS for 2 weeks at 4 °C. Arches were embedded in 1.5% agarose and scanned at 5 µm resolution (70 kV and 142 µA with no filter) using a SKYscanner 1172 scanner (Bruker, Coventry, UK). Raw files were reconstructed using NRecon (Bruker).

### Statistical analysis

Data are presented as mean ± SEM. Normality was tested using the Shapiro–Wilk test. Groups were compared using the Mann–Whitney U test for non-parametric data or an un-paired Student’s *t* test for parametric data. When comparing multiple groups data were analysed by analysis of variance (ANOVA) with Newman–Keuls post-test for parametric data or Kruskal–Wallis test with Dunns post-test for non-parametric data. When more than two independent variables were present a two-way ANOVA with Tukey’s multiple comparisons test was used. A value of *P* < 0.05 was considered statistically significant. All experiments and analysis were carried out by personnel blinded to genotype. The experimental unit was defined as a single animal, animals of both genotypes were caged together and in all experiments animals of both genotypes were derived from more than one cage. Age matched mice were randomly assigned to experiments.

## Supplementary Information

Below is the link to the electronic supplementary material.

Atherosclerosis regression resulted in a stable plaque phenotype in aortic roots with increased collagen and decreased macrophage content. a Representative images of aortic roots, macrophages (Galectin-3 positive area, stain red). b A significant increase in collage content (as a percentage of plaque area) was observed after pI:pC treatment to induce atherosclerosis regression in both genotypes (*=P<0.05 two way ANOVA, comparing pI:pC treatment with respective controls). However, no difference was observed between genotypes (P>0.05 two way ANOVA, comparing between genotypes). c A significant decrease in macrophage content was observed after pI:pC treatment to induce atherosclerosis regression in both genotypes (*P≤0.05 two way ANOVA, comparing pI:pC treatment with respective controls). However, no difference was observed between genotypes (P>0.05 two way ANOVA, comparing between genotypes). Data are expressed as the mean±SEM, with each data point representing an individual mouse. Black symbols = Abcg1+/+ REVERSA, grey symbols = Abcg1-/- REVERSA. Open symbols atherosclerosis progression study harvested at 16 weeks of age. Closed symbol regression study harvested at 20 weeks of age 4 weeks after pI:pC injection. (PDF 298 KB)

## Data Availability

All data used to generate the results reported in this paper are available from request from the authors.
