# Supplementary figures and images for: Mutagenesis on a complex mouse genetic background by site-specific nucleases
Source: Transgenic Res. 2024 Aug 1;33(5):415–26. doi: 10.1007/s11248-024-00399-5 (PMC11588839; doi:10.1007/s11248-024-00399-5)

a

Non reversed

Reversed

*Abcg1*<sup>+/+</sup>*Abcg1*<sup>-/-</sup>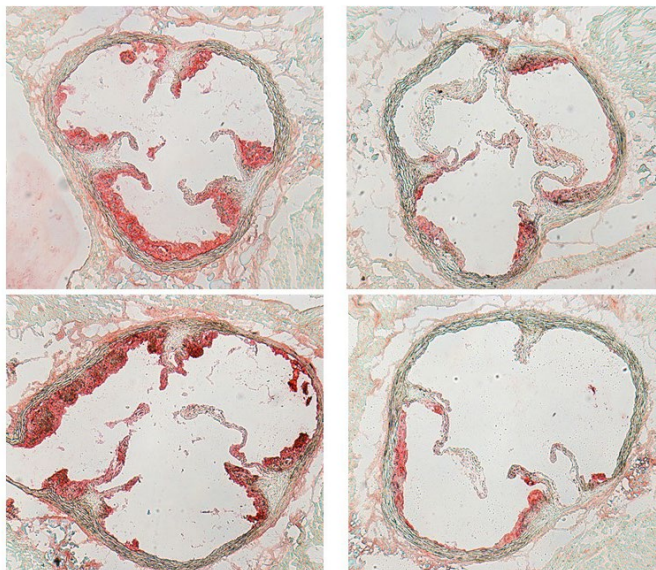

b

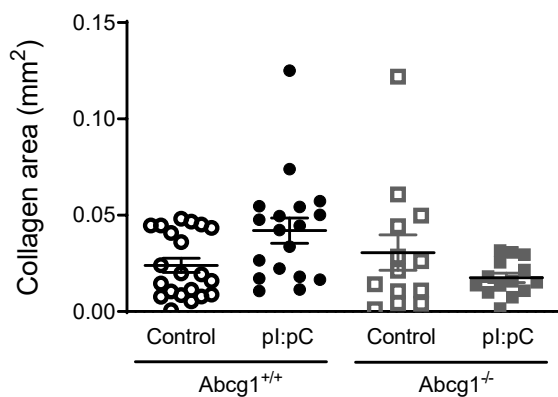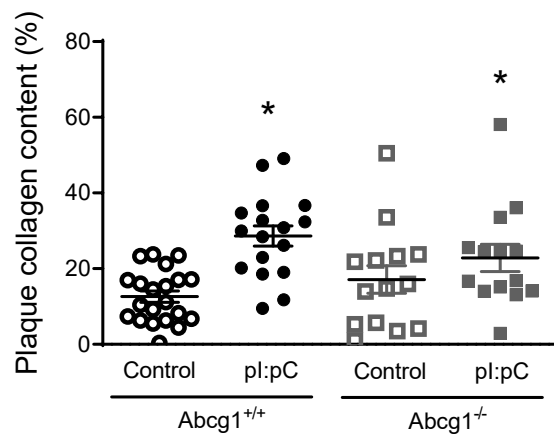

c

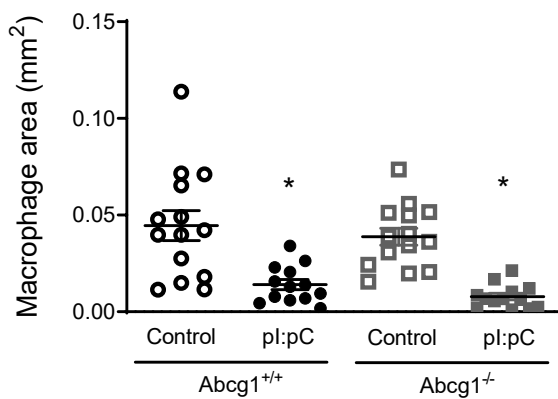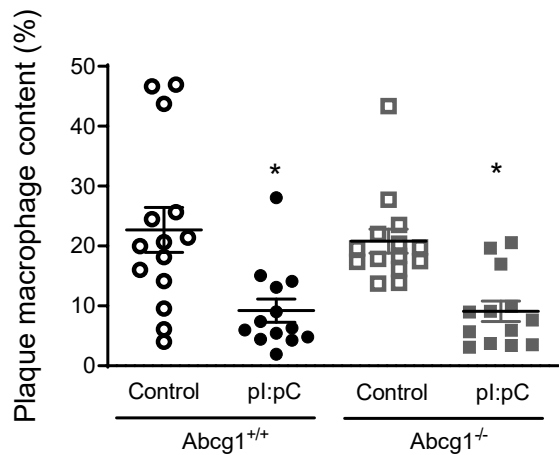

Supplement: Supplementary file 1 — Atherosclerosis regression resulted in a stable plaque phenotype in aortic roots with increased collagen and decreased macrophage content. a Representative images of aortic roots, macrophages (Galectin-3 positive area, stain red). b A significant increase in collage content (as a percentage of plaque area) was observed after pI:pC treatment to induce atherosclerosis regression in both genotypes (*=P<0.05 two way ANOVA, comparing pI:pC treatment with respective controls). However, no difference was observed between genotypes (P>0.05 two way ANOVA, comparing between genotypes). c A significant decrease in macrophage content was observed after pI:pC treatment to induce atherosclerosis regression in both genotypes (*P≤0.05 two way ANOVA, comparing pI:pC treatment with respective controls). However, no difference was observed between genotypes (P>0.05 two way ANOVA, comparing between genotypes). Data are expressed as the mean±SEM, with each data point representing an individual mouse. Black symbols = Abcg1+/+ REVERSA, grey symbols = Abcg1-/- REVERSA. Open symbols atherosclerosis progression study harvested at 16 weeks of age. Closed symbol regression study harvested at 20 weeks of age 4 weeks after pI:pC injection. (PDF 298 KB) [file 11248_2024_399_MOESM1_ESM.pdf]
